# Analysis of the main indicators and risk factors of ultrasonic shear wave elastography for the diagnosis of osteoarthritis among adults

**DOI:** 10.3389/fmed.2024.1366793

**Published:** 2024-03-14

**Authors:** Jiong Zhang, Xiaozuo Zheng, Ying Zhao

**Affiliations:** ^1^Department of Ultrasound, The Third Hospital of Hebei Medical University, Shijiazhuang, China; ^2^Department of Osteoarthritis, The Third Hospital of Hebei Medical University, Shijiazhuang, China

**Keywords:** ultrasonic shear wave elastography, ultrasound, osteoarthritis, adults, diagnosis

## Abstract

**Objective:**

This study was conducted to explore the main indicators of ultrasonic shear wave elastography (SWE) for the diagnosis of osteoarthritis (OA) and its influencing factors.

**Methods:**

We collected 910 patients between January 2018 and November 2023 from the department of ultrasound, Third Hospital of Hebei Medical University. Logistic regression was used to analyze the effects of age, gender, body mass index (BMI), smoking, alcohol, hypertension and diabetes on the diagnosis of OA by SWE.

**Results:**

The results showed that medial meniscal projection distance (MMPD) and OA had a positively correlated dose–response relationship (OR = 2.12, 95%CI (1.53, 3.95), trend *p* < 0.05). Also, medial meniscus elastometry (MME) had a positive dose–response correlation with OA (OR = 8.98, 95%CI (3.89, 11.52), trend *p* < 0.05). In addition, regarding the analysis of factors influencing the diagnosis of OA, the risk of OA was significantly higher in the older age group [OR = 1.11, 95%CI (1.01, 1.25)], and the risk of diagnosis in OA was high in the high BMI group [OR = 1.8, 95%CI (1.23, 3.01)].

**Conclusion:**

In diagnosing OA, MMPD and MME can be used as reliable indicators, while people of advanced age and high BMI have a high possibility diagnosed with OA.

## Introduction

Osteoarthritis (OA) is a chronic joint disease that is highly prevalent worldwide and a whole joint disease involving all joint tissues such as menisci, subchondral bone, infrapatellar fat pad and synovial membrane (1, 2). The 2015 World Health Organization Global Report on Aging and Health highlights OA as the leading cause of disability in adults aged more than 60 (3–5). Globally, the proportion of joint-related disability due to OA increased by 114.5% from 1990 to 2019. Given the aggravating trend of global population aging and the continued increase in the prevalence of OA, global healthcare expenditure is also expected to be huge in the future (6, 7).

The most common positions affected by OA are the hip, knee and hand. The knee is the most frequently affected joint, and knee OA is associated with excessive joint loading and injury (8, 9). X-ray radiography is frequently used for the diagnosis of OA, but x-ray is an indirect and insensitive way of detecting osteoarticular cartilage (10). Arthroscopy, although a reliable and sensitive method, is an invasive technique (11). Magnetic resonance imaging (MRI) is a non-invasive method that provides multiplanar imaging, and excellent soft tissue contrast, however, it is expensive and time consuming (12). Ultrasound (US) is a reliable tool for assessing the integrity and thickness of articular cartilage. Its advantages include being inexpensive, non-invasive, rapid and easily accessible (13).

Ultrasonic shear wave elastography (SWE) is a recently developed technique that is based on the measurement of shear wave velocity generated by ultrasound pulses (13). The technique can be used to assess tissue elasticity. SWE was first applied in breast lesions and thyroid gland lesions, and was subsequently used to assess liver fibrosis (14). More recently, SWE has also been used to assess the mechanical properties of musculoskeletal tissues (12). The SWE technique identifies differences in stiffness between tissues, which is a good indicator of damage to articular cartilage.

Although elastic ultrasound has been previously reported to be useful in the diagnosis of OA (11, 13), there is limited research on the effective indicators of OA diagnosis in SWE and their risk factors, so we conducted this study.

## Methods

### Patients selection

The data in this study were obtained from the Department of Ultrasound, Third Affiliated Hospital of Hebei Medical University. We obtained raw data from January 2018 to November 2023 for 910 patients, including 210 patients with OA and 700 samples without OA. The data of this study were approved by the Ethics Committee from the Third Affiliated Hospital of Hebei Medical University (No. 1354064). All patients/subjects signed the informed consent and that the study was performed in accordance with the ethical standards of the 1964 Declaration of Helsinki as revised in 2013. The patient inclusion and exclusion flow chart was shown in Supplementary Figure S1. The main inclusion criteria were as follow: (a) Adults patients ≥20 years old receiving ultrasonic shear wave elastography; (b) Patients had eligible health and diagnosis criteria; (c) Patients did not receive any surgery; (d) Patients agree to participate in the study. The main exclusion criteria were as follow: (a) Patients with meniscal injuries (based on MRI evaluation); (b) Patients lack information of diagnosis or other covariates; (c) Patients do not want to join in the study.

### Definition of OA

The typical diagnosis of OA include (1): (A) Joint pain: It is mostly a slight dull pain at rest, while a little bit of activity can reduce the pain. (B) Joint swelling: Most of the patients may have joint swelling, bleeding or fluid. (C) Joint stiffness: Joints become inflexible and stiff when they are at rest, and stiffness occurs when they wake up or sit upright. (D) Restriction of activities: With the development of the disease, patients gradually develop muscle atrophy and joint deformity around the joints, resulting in dysfunction. (E) Bone friction sensation: When the articular cartilage is destroyed and the joint surface is incomplete, the joint activities will have the feeling of joint friction or creaking.

### Ultrasonic shear wave elastography

**Figure 1:**
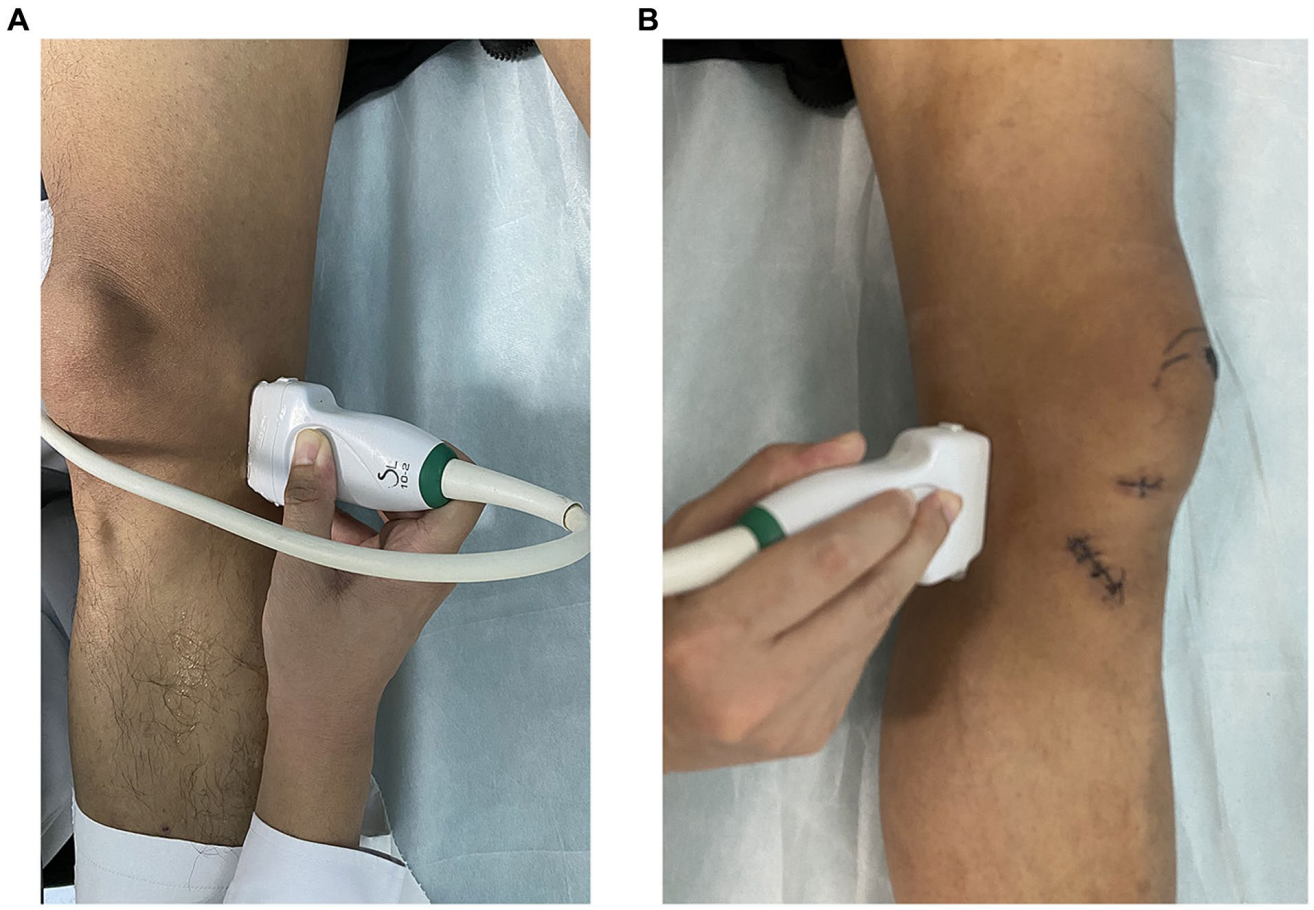
Ultrasonic shear wave elastography operation. **(A)** Ultrasound measurement of meniscus in a supine position with knee joint in an unloaded state. **(B)** Measurement of meniscus position in supine position using ultrasound in patients after knee joint surgery.

SWE were performed using an Aixplorer ultrasound machine equipped with a 12 MHz shallow linear transducer (SuperSonic Imagine, France). The first set of two ultrasound images was obtained in the flat lying position when the subject model was in the flat lying position with the ultrasound probe located in the position (Figure 1A), and then another set of two ultrasound images was obtained in the bipedal natural standing position. When the ultrasound probe was located in the position from anterior angle (Figure 1B). The medial meniscus of the knee was shown in the lying and standing positions, at which point the images were frozen and the line connecting the medial femoral cortex to the tibial cortex was immediately drawn using the electronic caliper function on the ultrasound machine. The distance of the MME over the tibiofemoral line was measured using the electronic caliper function on the ultrasound machine, while the SWE function on the ultrasound machine was applied to measure the elasticity values associated with the medial meniscus.

**Figure 2:**
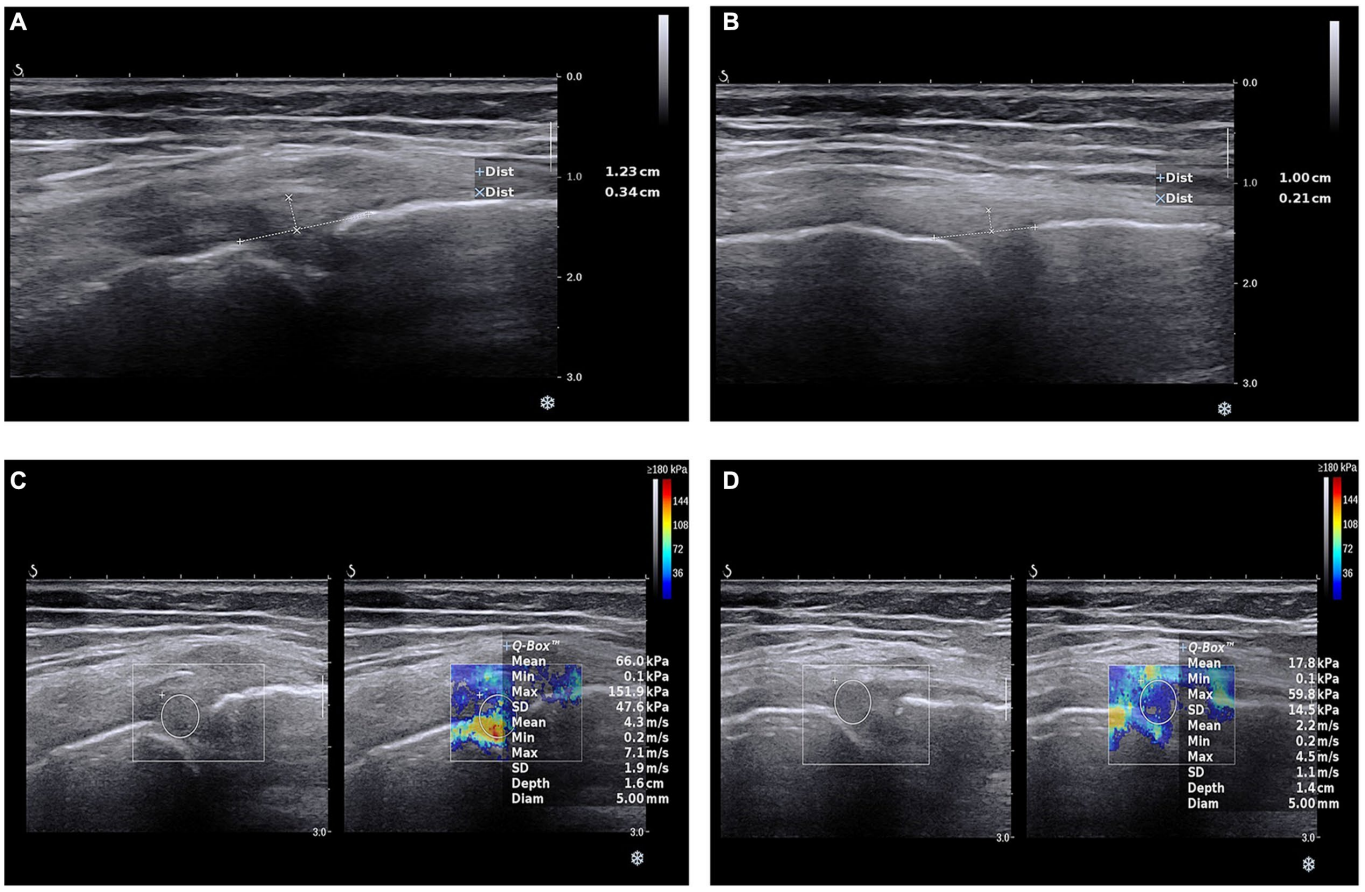
Ultrasonic shear wave elastography images. **(A)** Medial meniscal projection distance in osteoarthritis patients. **(B)** Medial meniscal projection distance in health people. **(C)** Medial meniscus elasticity in osteoarthritis patients. **(D)** Medial meniscus elasticity in health people.

During the scanning process, the angle of the probe was adjusted so that the incident sound wave was perpendicular to the surface of the articular cartilage as much as possible. Finally, we collected the medial meniscal projection distance (MMPD) and the medial meniscus elasticity (MME). The operation of SWE is shown in Figure 1. In addition, the images for MMPD and MME in OA diagnosis and a healthy control is shown in Figure 2.

### Covariates

To resilient the SWE main indicator for OA, we also retrospectively collected data on participants’ age, gender, BMI, smoking, alcohol consumption, hypertension, and diabetes from the hospital’s electronic medical record system.

### Logistic regression

To analyze whether there was a dose–response association between MMPD, MME and OA with different covariates, we used stepwise logistic regression (15). There were 4 models including, model 1 = no adjustment, model 2 = model 1 plus sex, age (years, continuous), and BMI, model 3 = model 2 plus smoking and alcohol, and model 4 = model 3 plus hypertension and diabetes. The effects of each covariate on OA were further analyzed by multifactor logistic regression and forest plots were drawn. The MMPD and MME were orderly cut into 4 quartiles and 2–4 quartiles were compared with quartile 1 to assess the dose response relationship between MMPD, MME and OA. Odds ratio (OR) was the main indicator to assess the associations, and OR < 1 represented negative relationship with OR > 1 represented positive relationship, meanwhile OR = 1 standed for meaningless.

The R software 4.1.2 (The R Foundation for Statistical Computing, United States) was used to perform all of the aforementioned analysis. *p* < 0.05 on both sides was regarded as statistically significant.

## Results

Based on the presence or absence of OA, we analyzed the baseline data. As shown in Table 1, there were differences between the two groups in age, gender, BMI, and smoking and alcohol, MMPD and MME. While there was no difference in hypertension and diabetes (Table 1).

**Table 1:** Characteristics of included participants.

	NoOA (*N* = 700)	OA (*N* = 210)	*p*
**Age**	39.1 (19.2)	53.1 (7.87)	<0.001
**Gender**			<0.001
female	100 (14.3%)	140 (66.7%)	
male	600 (85.7%)	70 (33.3%)	
**BMI**	24.5 (3.83)	28.8 (3.16)	<0.001
**Smoking**			0.005
No	300 (42.9%)	170 (81.0%)	
Yes	400 (57.1%)	40 (19.0%)	
**Alcohol**			0.015
No	300 (42.9%)	160 (76.2%)	
Yes	400 (57.1%)	50 (23.8%)	
**Hypertension**			0.367
No	400 (57.1%)	90 (42.9%)	
Yes	300 (42.9%)	120 (57.1%)	
**Diabetes**			0.647
No	460 (65.7%)	120 (57.1%)	
Yes	240 (34.3%)	90 (42.9%)	
**MMPD(mm)**	2.31 (0.54)	4.42 (0.93)	<0.001
**MME(Kp)**	28.9 (8.44)	48.2 (14.0)	<0.001

Using the Q1 group as a reference, we performed stepwise logistic regression (Table 2) to analyze whether the risk of OA changed with increasing SWE indicators in the Q2–Q4 groups. It can be seen in model 1, when only MMPD was used as the independent variable, there was a positive correlation dose–response relationship between this value and OA (trend *p* < 0.05) and the OR for Q4 was much greater than 1, [OR = 5.97, 95% CI (2.81, 4.98)], implying that the higher the level of MMPD, the higher the probability of having OA.

**Table 2:** Association between MMPD and OA.

	Model 1	Model 2	Model 3	Model 4
Q1	Reference	Reference	Reference	Reference
Q2	1.09 (0.34,2.72)	2.48 (0.56,3.25)	8.52 (0.12,11.58)	2.83 (0.52,3.89)
Q3	3.14 (2.01,4.82)	5.21 (1.28,6.89)	6.53 (0.79,9.89)	7.77 (0.68,10.84)
Q4	5.97 (2.81,4.98)	6.14 (2.33,7.59)	4.12 (2.58,6.58)	2.12 (1.53,3.95)
*P* for trend	<0.05	<0.05	>0.05	>0.05

Model 2 was constructed after demographic data were included as covariates and a positive correlation dose–response relationship remained. Model 3 had a positive correlation between high levels of MMPD and OA after lifestyle habits were put into the model [OR = 4.12, 95% CI (2.58, 6.58)], but there was no longer a dose–response relationship (Table 2). Model 4 also showed a positive association between high levels of MMPD and OA [OR = 2.12, 95% CI (1.53, 3.95)] after incorporating history of illness.

We also performed an analysis of MME and OA, which showed a dose–response relationship for all models 1 to 4 (trend *p* < 0.05). Moreover, with the gradual inclusion of the variables, the ORs increased minutely, reaching an OR of 8.98 [OR = 8.98, 95% CI (3.89, 11.52)] for Q4 in model 4 (Table 3).

**Table 3:** Association between MME and OA.

	Model 1	Model 2	Model 3	Model 4
Q1	Reference	Reference	Reference	Reference
Q2	1.16 (1.01,2.58)	3.31 (0.48,4.58)	6.29 (0.84,9.84)	6.89 (0.15,8.85)
Q3	2.31 (1.58,3.89)	9.53 (0.59,12.94)	3.39 (1.95,4.85)	4.58 (2.58,6.55)
Q4	1.73 (1.25,2.74)	1.81 (1.21,2.15)	7.76 (2.89,10.58)	8.98 (3.89,11.52)
*P* for trend	<0.05	<0.05	<0.05	<0.05

**Figure 3:**
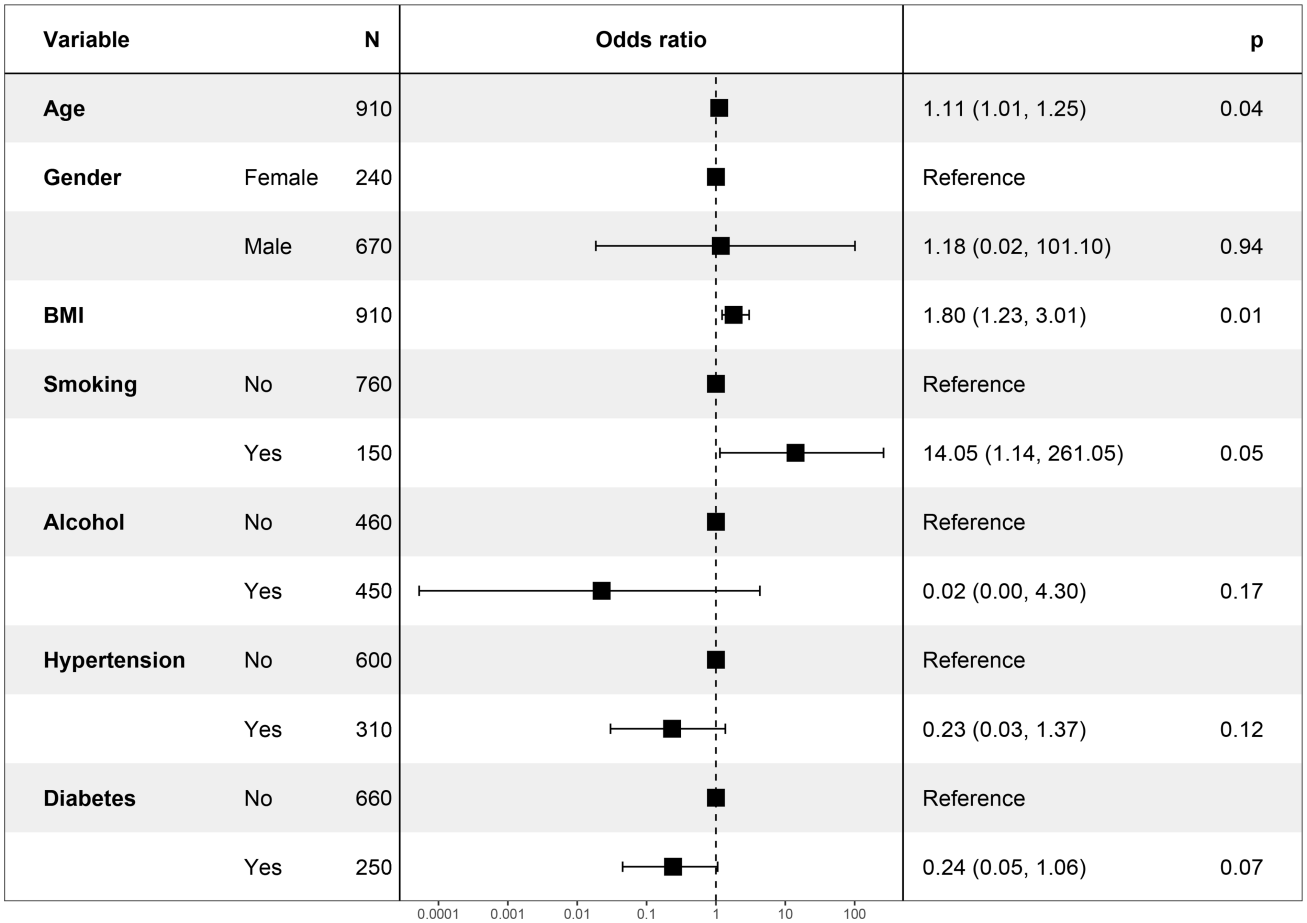
Forest plot of association between covariates and OA.

In addition to analysing the relationship between elastic ultrasound measurements and OA, we also analyzed the relationship between the included covariates and OA. As shown in Figure 1, the risk of OA was significantly higher in the older age group [1.11, 95% CI (1.01, 1.25)] and the risk of diagnosing OA was high in the high BMI group [1.8, 95% CI (1.23, 3.01)] (Figure 3).

## Discussion

In this study, we assessed knee OA lesions by using two metrics including MMPD and MME. We found that the correlation between the two measurements for assessing knee OA was high. Subsequently, exploring the factors influencing the SWE measurement of OA, people with advanced age and high BMI had a higher probability of being diagnosed with OA.

MME is strongly associated with knee OA and meniscal tears, which have also been described as radial displacement of the medial meniscus or medial radial displacement of the medial meniscus (16). Longitudinal studies have shown that the value of MME increases with increasing Kellgren/Lawrence radiological degrees in both supine and standing assessments of the knee (17). Similarly, studies have confirmed a significant correlation between an MME greater than or equal to 3 mm on MRI and an increase in the severity of cartilage damage, and a close association between MME and joint space narrowing (18). MME has been shown to be an independent predictor of disease progression in OA and is strongly associated with tibial and femoral cartilage loss (19).

However, there are limited studies on MMPD for the diagnosis of OA, and our study also supports the validity of MMPD for the diagnosis and prediction of OA, which needs to be confirmed by more relevant studies.

Previous reports have stated that there is a significant correlation between age and tendon, muscle and joint stiffness (20–22). This study also found a significant correlation between age and OA, while the correlation between degeneration and stiffness was equally significant. In moderate to severe OA, the meniscal matrix showed severe fibrocartilage detachment and extensive wear and tear, with reduced cellularity and cellular hypertrophy. Degeneration of the meniscus in OA is also found in people with high BMI, as evidenced by the accumulation of meniscal proteoglycans (23–25). These micro and macro changes in the degenerative meniscus are reflected by increased tissue elastic stiffness values measured by SWE (26, 27). Several previous researches supported that shear wave elastography can serve as an adjunctive tool to aid in the assessment of knee meniscal elasticity, especially can detect meniscus pathologies that may develop due to obesity and aging (28–30). These findings were totally consistent with ours. Ultrasound evaluation with SWE is an easily accessible, inexpensive imaging modality that also provides real-time dynamic evaluation and guides percutaneous interventional procedures. However, evaluation of the meniscus by ultrasound and SWE may be technically difficult due to adjacent bone and ligamentous structures and narrowing of the joint space due to degenerative disease (31).

In conclusion, these preliminary findings suggest that MME and MMPD correlate with the degree of meniscal degeneration, which is seen to reflect knee OA lesions. So, it is reasonable to assume that SWE could be used as a potential tool for assessing OA, especially in aging and high BMI people.

There are several limitations of this study. Firstly, the sample size was small, which reduced the reliability and validity. Secondly, there was a lack of low-age samples. Thirdly, this study only involved measurements of the medial meniscus of the knee and lacked values for the lateral meniscus and other joint measurements. Further studies are needed to improve the reliability of our findings.

## Data availability statement

The raw data supporting the conclusions of this article will be made available by the authors, without undue reservation.

## Ethics statement

The studies involving humans were approved by this study were approved by the Ethics Committee from the Third Affiliated Hospital of Hebei Medical University (No. 1354064). The studies were conducted in accordance with the local legislation and institutional requirements. The participants provided their written informed consent to participate in this study.

## Author contributions

JZ: Data curation, Formal analysis, Investigation, Writing – original draft. XZ: Methodology, Software, Writing – original draft. YZ: Conceptualization, Formal analysis, Methodology, Validation, Writing – review & editing.
