# Supplementary figures and images for: Analysis of the main indicators and risk factors of ultrasonic shear wave elastography for the diagnosis of osteoarthritis among adults
Source: Front Med (Lausanne). 2024 Mar 14;11:1366793. doi: 10.3389/fmed.2024.1366793 (PMC10972896; doi:10.3389/fmed.2024.1366793)

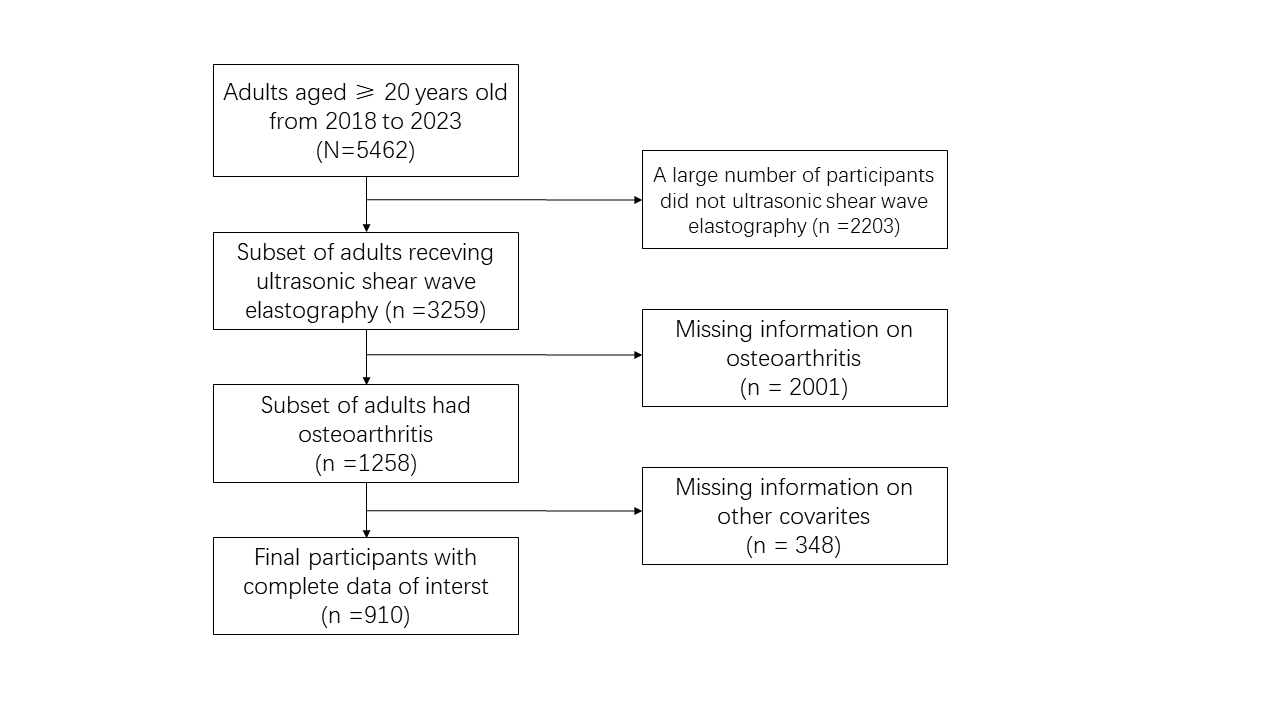

Supplement: Supplementary file 1 [file Image_1.TIF]
